# MRI-derived tumor volume vs. pathological tumor diameter: a comparative study of prognostic value for disease-free survival in multifocal/multicentric breast cancer

**DOI:** 10.3389/fonc.2026.1749057

**Published:** 2026-04-17

**Authors:** Jiangni Song, Juan Mao, Nianyang Li, Yue Yin, Susu Yang, Yitao Liu, Ziyi Yu, Kaiwen Liu, Shouju Wang, Yi Zhao

**Affiliations:** 1 Department of Breast Surgery, The First Affiliated Hospital of Nanjing Medical University, Nanjing, Jiangsu, China; 2 Department of Health Promotion Center, Affiliated Hospital of Yangzhou University, Yangzhou University, Yangzhou, Jiangsu, China; 3 Department of Radiology, The First Affiliated Hospital of Nanjing Medical University, Nanjing, Jiangsu, China

**Keywords:** Breast cancer, MRI, multicentric, multifocal, tumor volume, tumor diameter

## Abstract

**Background and objective:**

Multifocal/multicentric breast cancer (MMBC) is characterized by multiple tumor foci in the same breast, but conventional T staging based on the largest lesion (Dmax) may underestimate true tumor burden. This study aimed to evaluate whether MRI-derived tumor volumes (Vmax and Vsum) provide superior prognostic value for disease-free survival (DFS) compared with pathological tumor diameters (Dmax and Dsum).

**Materials and methods:**

We retrospectively reviewed 111 patients with MMBC treated between January 2015 and June 2020. Tumor volumes were quantified from preoperative 3T MRI, and pathological diameters and clinicopathological variables were collected from medical records. To normalize distributions, tumor size metrics were log_2_-transformed. Associations with DFS were assessed using Cox regression, and predictive performance was evaluated via Kaplan–Meier curves, receiver operating characteristic (ROC) analysis, and decision curve analysis (DCA).

**Results:**

Log_2_-transformed tumor size metrics, positive lymph node count, and molecular subtype were significant prognostic factors for DFS (all P < 0.05). Volumetric metrics provided stronger risk stratification than diameter-based metrics (log-rank P < 0.001). A multivariate Cox model combining positive lymph node count with log_2_(Vsum) showed the best predictive performance (AIC = 138.42; C-index = 0.845) and remained robust after adjusting for molecular subtype and Ki-67. This model achieved higher sensitivity for 5-year DFS prediction (0.923 vs 0.769 for log_2_(Dmax)), and DCA demonstrated greater net clinical benefit at threshold probabilities below 15.5%.

**Conclusions:**

MRI-derived sum of tumor volumes (Vsum) provides superior prognostic value for DFS in MMBC compared with diameter-based metrics. Integrating Vsum with lymph node burden may improve risk stratification and support personalized treatment planning.

## 1 Introduction

Breast cancer remains the most prevalent malignancy among women worldwide, with over 2.3 million new cases annually, representing 11.6% of all cancers (1, 2). Multifocal/multicentric breast cancer (MMBC) constitutes a distinct subtype. Typically, multifocal breast cancer (MFBC) is defined as the presence of two or more independent lesions within the same quadrant, whereas multicentric breast cancer (MCBC) refers to lesions located in different quadrants (3–5). Owing to the absence of a standardized definition and differences in imaging techniques, the reported incidence of MMBC varies widely, ranging from 1% to 77% of all breast cancers (4–7). In this study, multifocal and multicentric cases were not distinguished.

Compared with unifocal breast cancer, MMBC is associated with higher rates of lymph node metastasis, increased risk of local recurrence, and poorer long-term outcomes (8–10). Previous studies have reported that approximately 15%–30% of MMBC cases exhibit discordance in molecular subtype or clonal composition among different tumor foci, reflecting substantial intrapatient heterogeneity (4, 11–13). This tumor heterogeneity may represent underlying biological diversity. Furthermore, emerging evidence suggests that both intralesional and interlesional heterogeneity in MMBC may be associated with metabolic reprogramming, including alterations in lipid, glucose, and amino acid metabolism, which in turn influence tumor growth, metastatic potential, and treatment response (14, 15). Similar phenomena have also been observed in other malignancies, such as neuroendocrine neoplasms, where metabolic heterogeneity has been proposed as a potential therapeutic target (16). Therefore, accurate assessment of tumor burden is essential for therapeutic decision-making and prognostic stratification. Because of this heterogeneity, reliance on the largest lesion alone may be insufficient.

Since the adoption of the tumor-node-metastasis (TNM) system, tumor size has remained a cornerstone of staging, guiding both treatment and prognosis. With advances in mammography, ultrasound, and dynamic contrast-enhanced magnetic resonance imaging (DCE-MRI), an increasing number of patients are diagnosed before regional lymph node or distant metastasis occurs (17, 18). Under these circumstances, the T stage of the primary tumor plays a decisive role. According to the 8th edition of the AJCC Breast Cancer Staging Manual, the T stage of MMBC is based on the maximum diameter of the largest lesion (Dmax), with the suffix “(m)” used to indicate multifocal or multicentric characteristics (19). However, recent studies suggest that the sum of all lesion diameters (Dsum) may better represent overall tumor burden (4, 20, 21). Yet, both Dmax and Dsum are one-dimensional metrics that fail to capture the true three-dimensional nature of tumors, making accurate assessment of MMBC burden a persistent challenge.

Given the limitations of diameter-based metrics in capturing the overall tumor burden of MMBC, alternative quantitative approaches have been explored. With the increasing use of breast MRI, MRI-based volumetric measurement has emerged as a promising approach for quantifying tumor burden. In various solid tumors, such as prostate and endometrial cancers, imaging-based tumor volume has demonstrated superior prognostic value over diameter-based measures (22–26). In breast cancer, particularly in neoadjuvant therapy studies, volumetric changes have shown strong correlations with prognosis (27–29). However, despite these advances, the prognostic value of volumetric parameters in MMBC has not been systematically evaluated. In particular, whether maximum tumor volume and the sum of tumor volumes provide superior predictive performance compared with conventional diameter-based metrics remains unclear, representing an important gap in current staging and risk stratification.

To address this gap, this study included 111 patients with MMBC, with a median follow-up of 77 months, to systematically compare MRI-derived tumor volume parameters with traditional pathological diameter metrics in predicting disease-free survival (DFS). Specifically, we comprehensively evaluated the prognostic performance of maximum diameter (Dmax), sum of diameters (Dsum), maximum tumor volume (Vmax), and sum of tumor volumes (Vsum), with particular emphasis on whether Vsum could outperform Dmax in prognostic prediction. Our study aims to provide evidence for improving the accuracy of staging and risk stratification in MMBC.

## 2 Materials and methods

### 2.1 Study population and case selection

**Figure 1:**
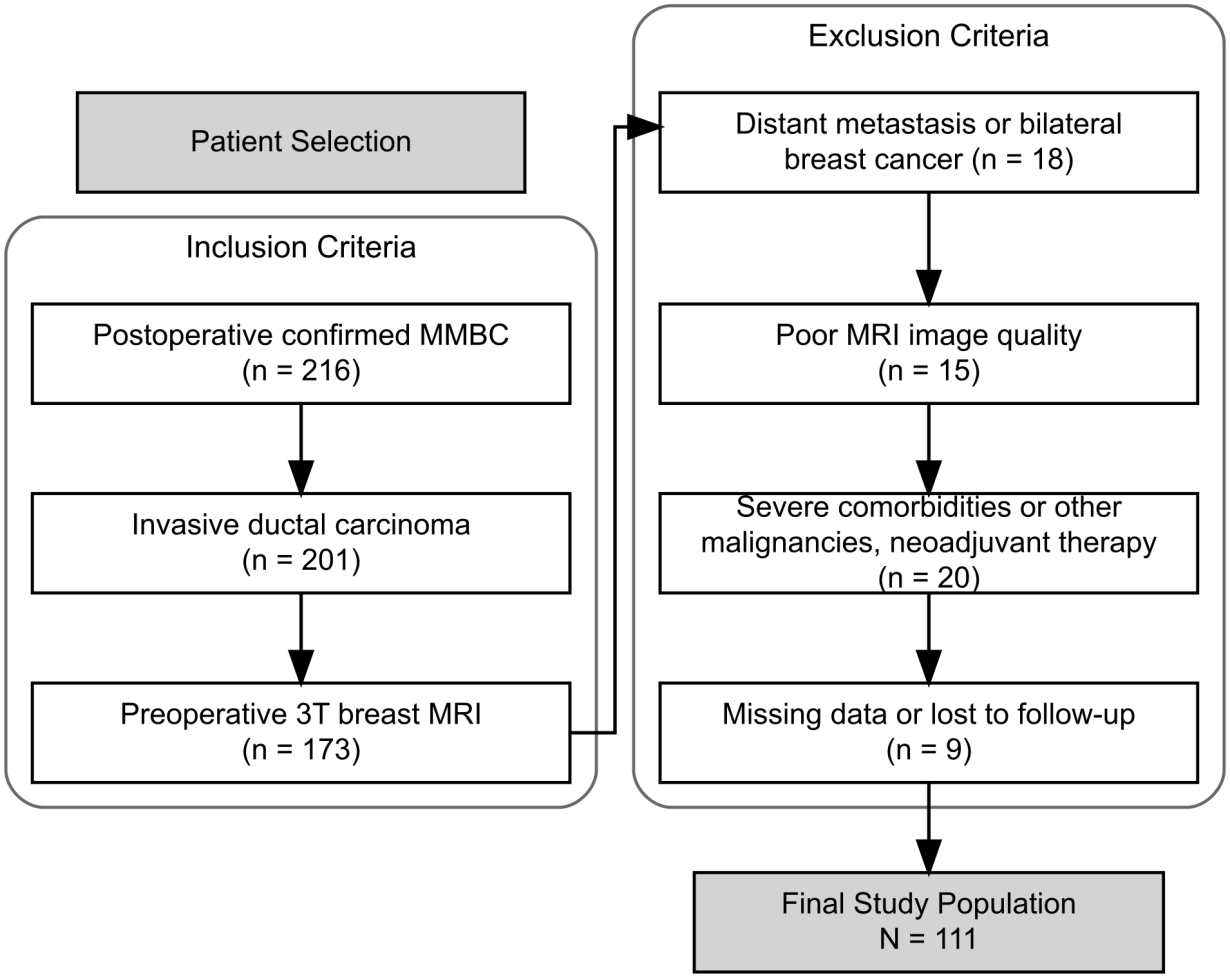
Patient selection flowchart.

To ensure adequate follow-up for survival analysis, we included patients treated at least 5 years before the final follow-up. We retrospectively reviewed the medical records of patients diagnosed with MMBC who underwent surgery between January 2015 and June 2020 at the Department of Breast Surgery, the First Affiliated Hospital of Nanjing Medical University. The patient selection flowchart is shown in **Figure 1**.

The inclusion criteria were as follows: (1) postoperative pathological confirmation of MMBC; (2) invasive ductal carcinoma (IDC) in all lesions; and (3) availability of preoperative 3T breast MRI.

The exclusion criteria were as follows: (1) bilateral breast cancer or distant metastasis at diagnosis; (2) poor-quality MRI images (e.g., motion artifacts or indistinct lesion margins); (3) severe comorbidities, other malignancies, or prior neoadjuvant therapy; and (4) incomplete clinicopathological information or loss to follow-up (defined as no documented visits for ≥12 months with unknown survival status).

A total of 111 eligible patients were included after applying these criteria. All patients received standardized treatment according to the Chinese Society of Clinical Oncology (CSCO) guidelines in effect at that time.

This study was approved by the Institutional Review Board of the First Affiliated Hospital of Nanjing Medical University (approval number: 2025-SR-1223), and the requirement for informed consent was waived due to the retrospective nature of the study.

### 2.2 Clinical and pathological data collection

Clinical and pathological data were collected independently by two researchers. The data were then cross-checked for consistency, and any discrepancies were resolved through consensus with a senior pathologist and a breast surgeon. These data were obtained from electronic medical records and pathology archives, including age, number of lesions, number of positive lymph nodes (positive LN count), nodal status, histologic grade, lymphovascular/perineural invasion (LVI), and surgical approach. Immunohistochemical (IHC) markers included estrogen receptor (ER), progesterone receptor (PR), human epidermal growth factor receptor 2 (HER2), Ki-67, and fluorescence *in situ* hybridization (FISH) results.

Molecular subtypes were categorized as HR+/HER2−, HR+/HER2+, HR−/HER2+, and HR−/HER2− (triple-negative) based on the following criteria: ER or PR positivity was defined as ≥1% nuclear staining; HER2 positivity as an IHC score of 3+ or 2+ with HER2 gene amplification by FISH; and Ki-67≥20% as high proliferation. According to the mainstream clinical guidelines at that time, only the largest lesion in MMBC was emphasized for immunohistochemical assessment. Therefore, not all lesions underwent IHC analysis. Specifically, IHC analysis was performed for all lesions in 45 of 111 patients (40.5%), whereas in the remaining 66 patients (59.5%), only the dominant (largest) lesion was assessed. When multiple lesions differed in histologic grade, receptor status, or Ki-67 expression, the highest grade, receptor-positive status, or highest Ki-67 value among the lesions was used to represent overall disease characteristics, to reflect overall tumor aggressiveness.

### 2.3 Measurement of tumor size metrics

Pathological tumor diameters were obtained from postoperative pathology reports. The maximum diameter (Dmax) was defined as the largest dimension of the dominant lesion, while the sum of diameters (Dsum) represented the cumulative maximum diameters of all independent lesions (precision: 0.1 cm).

**Figure 2:**
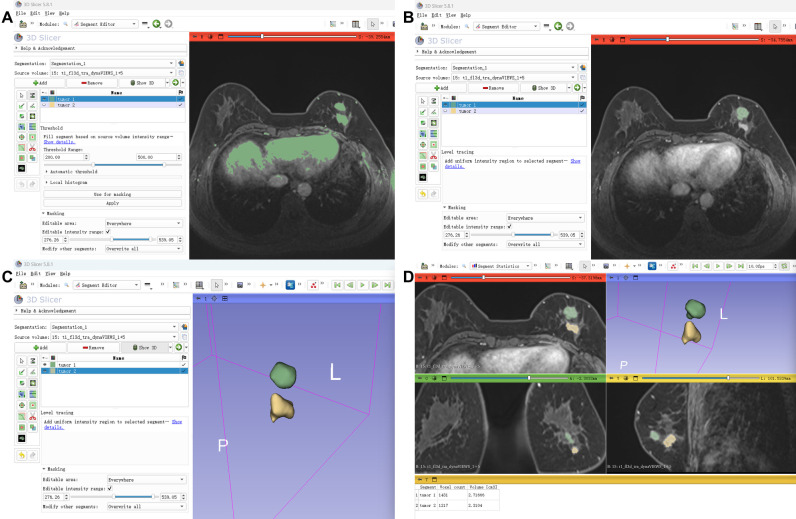
Tumor 3D volume measurement using 3D Slicer software. **(A)** DICOM images were imported and segmented using the “Threshold” tool. **(B)** Tumor boundaries were delineated using the “Level Tracing” tool, excluding non-tumor tissues. **(C)** The “Show 3D” module generated a three-dimensional reconstruction of the tumor’s spatial structure. **(D)** Tumor volume was calculated automatically using the “Segment Statistics” module. Three-dimensional tumor models were then generated (example: 2.7167 cm³ and 2.3104 cm³).

MRI-derived tumor volumes were obtained from preoperative 3.0T dynamic contrast-enhanced breast MRI (SIEMENS 3.0T MAGNETOM Trio Tim System). Imaging sequences included axial T1-weighted, T2-weighted, diffusion-weighted (DWI), and multiphase dynamic contrast-enhanced (DCE) imaging, with a slice thickness of 0.9 mm. A gadolinium-based contrast agent (gadopentetate dimeglumine) was administered at 0.1 mmol/kg at a rate of 2 ml/s. Original MRI DICOM files were imported into 3D Slicer (version 5.8.1) for subsequent image processing and volumetric analysis. The most conspicuous T1-weighted DCE phase was selected for tumor segmentation. A semi-automatic thresholding approach was applied, with the threshold (range: 200–500 in relative signal intensity units) determined empirically based on contrast-enhanced signal differences between tumor tissue and surrounding normal parenchyma. Manual refinement was subsequently performed to optimize lesion boundaries. Because MRI signal intensity is not standardized across patients, threshold selection was adjusted on a per-case basis. All readers were blinded to clinical outcomes during image analysis. Three-dimensional tumor models and corresponding volumes were then automatically generated (**Figure 2**). The maximum volume (Vmax) corresponded to the dominant lesion, while the sum of tumor volumes (Vsum) was defined as the total volume of all lesions (precision: 0.0001 cm³).

Not all MRI-detected enhancing lesions underwent separate preoperative biopsy; however, all lesions included in the volumetric analysis were confirmed as invasive ductal carcinoma (IDC) by postoperative pathological examination. Radiologic–pathologic correlation was performed to ensure spatial concordance between MRI enhancement and histologically confirmed carcinoma foci. All measurements were independently performed by one senior radiologist and two breast surgeons. The Dice similarity coefficient (0.854 ± 0.08) demonstrated good interobserver consistency. A two-way random-effects model further confirmed excellent interobserver reliability for both volumetric parameters, with Vmax yielding ICC (1, 2) = 0.939 (95% CI: 0.917–0.956, P < 0.001) and Vsum yielding ICC (1, 2) = 0.943 (95% CI: 0.922–0.959, P < 0.001). The final volume values represent the average across all observers.

### 2.4 Follow-up and outcome definition

Patients were followed through outpatient visits and telephone interviews. Follow-up assessments occurred every 3 months during the first 2 years, every 6 months from years 3–5, and annually thereafter. Routine evaluations included breast ultrasound or mammography, chest CT, abdominal imaging, and serum tumor markers (CA15–3 and CEA). Bone scans or PET-CT were performed when clinically indicated.

The primary endpoint was disease-free survival (DFS), defined as the time from surgery to the first documented event of recurrence, metastasis, second primary malignancy, or death from any cause. Follow-up was completed on September 1, 2025, with a median duration of 77 months (range: 8–128 months).

### 2.5 Statistical analysis

The normality of continuous variables was assessed using the Shapiro–Wilk test. Variables with a normal distribution are presented as mean ± standard deviation (mean ± SD), whereas non-normally distributed variables are expressed as median [IQR]. Tumor size metrics were log_2_-transformed to improve normality, and Spearman’s rank correlation was used to assess diameter–volume relationships before and after transformation. Associations between tumor size metrics and disease-free survival (DFS) were assessed using Cox proportional hazards models. Variables with P < 0.05 in univariable analysis, along with clinically relevant prognostic factors, were included in multivariable Cox regression models (one covariate plus one tumor-size variable). The proportional hazards assumption was tested using Schoenfeld residuals, with P > 0.05 indicating no violation. The robustness of the multivariable Cox model was evaluated through 1,000 bootstrap resamplings. Model performance was compared using the Akaike information criterion (AIC), concordance index (C-index), and likelihood ratio test (LRT). Patients were dichotomized according to the median values of log_2_(Dmax) and log_2_(Vsum). Kaplan–Meier survival curves were generated using the survminer package, and intergroup differences were evaluated with the log-rank test. For 5-year DFS as the endpoint, receiver operating characteristic (ROC) curves and area under the curve (AUC) values were calculated using the pROC package, with DeLong’s test applied for group comparisons. Sensitivity and specificity were determined based on the Youden index. Finally, decision curve analysis (DCA) was performed using the rmda package to assess the clinical utility of the constructed logistic regression models. All statistical tests were two-sided, and P < 0.05 was considered statistically significant.

## 3 Results

### 3.1 Patient characteristics

Among the 111 patients with MMBC, the mean age was 46.6 ± 10.2 years, with 62.2% (69/111) aged ≤50 years, indicating a relatively young cohort. The median pathological maximum tumor diameter was 2.20 cm, and the sum of diameters (Dsum) was 3.40 cm. On MRI, the median maximum and total tumor volumes were 2.85 cm³ and 3.91 cm³, respectively. Regarding clinicopathologic features, 63.1% (70/111) had lymph node metastasis (median: 1 positive lymph node). The predominant molecular subtype was HR+/HER2− (64.0%), followed by HR+/HER2+ (15.3%), HR−/HER2+ (15.3%), and triple-negative (5.4%). High Ki-67 expression (≥20%) was present in 79.3% of cases, histologic grade III in 58.6%, and lymphovascular/perineural invasion (LVI) in 44.1%. Most patients had 2 lesions (81, 73.0%), whereas 30 patients (27.0%) had more than 2 lesions. Mastectomy was the primary surgical approach (81.1%), and breast-conserving surgery was performed in 18.9% of patients (**Table 1**). Overall, the cohort was relatively young, with frequent lymph node involvement and predominance of the HR+/HER2− molecular subtype.

**Table 1:** Baseline clinical and pathological characteristics of MMBC patients.

Characteristic	Category	Overall (n = 111)
Age, mean (SD), years		46.60 ± 10.20
	≤50 years, n (%)	69 (62.2)
	>50 years, n (%)	42 (37.8)
Maximum diameter (Dmax), median [IQR], cm		2.20 [1.70-2.90]
Sum of diameters (Dsum), median [IQR], cm		3.40 [2.50-5.05]
Maximum volume (Vmax), median [IQR], cm³		2.85 [1.20-5.15]
Sum of volumes (Vsum), median [IQR], cm³		3.91 [1.50-6.42]
Number of lesions, n (%)	2	81(73.0)
	>2	30(27.0)
Positive LN count, median [IQR]		1.00 [0.00-5.00]
Lymph node status, n (%)	Positive	70 (63.1)
	Negative	41 (36.9)
Molecular subtype, n (%)	HR+/HER2−	71 (64.0)
	HR+/HER2+	17 (15.3)
	HR−/HER2+	17 (15.3)
	HR−/HER2−	6 (5.4)
Ki-67 index, n (%)	High (≥20%)	88 (79.3)
	Low (<20%)	23 (20.7)
Histological grade, n (%)	I–II	46 (41.4)
	III	65 (58.6)
LVI, n (%)	Present	49 (44.1)
	Absent	62 (55.9)
Surgery, n (%)	Mastectomy	90 (81.1)
	BCS	21 (18.9)

### 3.2 Log_2_ transformation and correlation preservation

**Figure 3:**
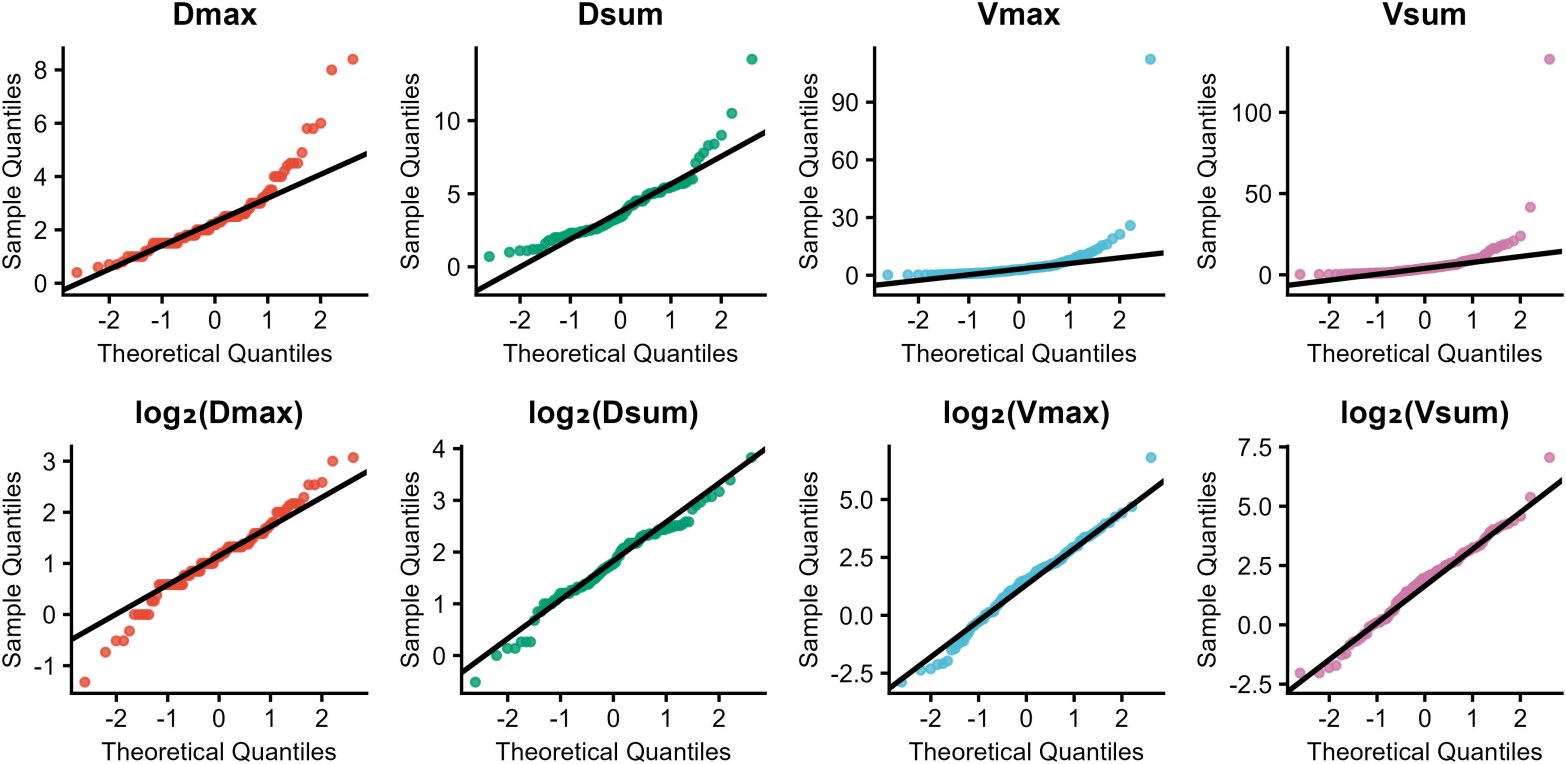
Quantile–Quantile (Q–Q) plots before and after log_2_ transformation of tumor size metrics.

The Shapiro–Wilk test indicated that all raw diameter and volume variables were right-skewed (P < 0.05). Log_2_ transformation significantly improved the normality of the tumor size variables. Specifically, the log_2_-transformed maximum tumor volume (log_2_(Vmax), W = 0.987, P = 0.354), sum of tumor volumes (log_2_(Vsum), W = 0.986, P = 0.282), and sum of tumor diameters (log_2_(Dsum), W = 0.979, P = 0.074) approximated a normal distribution. Additionally, the log_2_-transformed maximum tumor diameter (log_2_(Dmax), W = 0.976, P = 0.039) also demonstrated improved normality. Quantile–quantile (Q–Q) plots confirmed that these transformed variables aligned well with the theoretical normal distribution (**Figure 3**). Spearman’s rank correlation coefficients (r) remained unchanged before and after transformation (all P < 0.001), indicating that the transformation improved normality without affecting the relationships between variables (**Table 2**). Consequently, all subsequent analyses were performed using log_2_-transformed tumor size variables. These results show that log_2_ transformation normalizes tumor size variables while preserving their correlations.

**Table 2:** Spearman correlation coefficients between tumor size metrics.

Tumor metric comparison	Data transformation	n	r (95% Cl)	*P* value
Dmax vs. Vmax	Original	111	0.710(0.600-0.794)	<0.001
	log_2_-Transformed	111	0.710(0.600-0.794)	<0.001
Dsum vs. Vsum	Original	111	0.683 (0.565-0.773)	<0.001
	log_2_-Transformed	111	0.683 (0.565-0.773)	<0.001
Dmax vs. Vsum	Original	111	0.704(0.593-0.790)	<0.001
	log_2_-Transformed	111	0.704(0.593-0.790)	<0.001

### 3.3 Univariable Cox regression analysis

Univariate Cox regression analysis identified several significant predictors of disease-free survival (DFS). The log_2_-transformed tumor size metrics, including the maximum tumor diameter (log_2_(Dmax)), sum of tumor diameters (log_2_(Dsum)), maximum tumor volume (log_2_(Vmax)), and sum of tumor volumes (log_2_(Vsum)), were all significant predictors of DFS (all P < 0.001). The corresponding hazard ratios (HRs) were 3.36 (95% CI: 1.80–6.28), 3.30 (95% CI: 1.67–6.52), 1.79 (95% CI: 1.36–2.34), and 1.85 (95% CI: 1.41–2.43), respectively.

Positive lymph node (LN) count was also a strong prognostic factor for DFS (HR = 1.14, 95% CI: 1.08–1.22, P < 0.001). The number of tumors did not significantly affect DFS (HR = 0.99, 95% CI: 0.47–2.07, P = 0.98).

Molecular subtype significantly influenced DFS (P = 0.012), with the HR−/HER2+ subtype associated with the highest risk (HR = 5.73, 95% CI: 2.00–16.41, P = 0.001). In contrast, the HR+/HER2+ subtype (HR = 1.31, 95% CI: 0.27–6.30, P = 0.738) and HR−/HER2− subtype (HR = 4.45, 95% CI: 0.92–21.52, P = 0.063) showed no significant association with DFS.

Other factors, including age, histologic grade, lymphovascular/perineural invasion (LVI), Ki-67 index, surgery type, and lymph node status, were not significantly associated with DFS (P > 0.05) (**Table 3**).

**Table 3:** Univariate Cox regression analysis of disease-free survival (*P* values are based on likelihood ratio tests).

Variable	HR (95% CI)	*P* value
Demographics		
Age (per 1-year increase)	1.00 (0.95-1.04)	0.844
Primary Tumor Burden (Original)		
Dmax (per 1 cm increase)	1.50 (1.21-1.86)	0.002
Dsum (per 1 cm increase)	1.31 (1.14-1.51)	0.002
Vmax (per 1 cm³ increase)	1.03 (1.01-1.04)	0.022
Vsum (per 1 cm³ increase)	1.02 (1.01-1.04)	0.015
Primary Tumor Burden (log_2_-transformed)		
log_2_(Dmax)	3.36 (1.80-6.28)	<0.001
log_2_(Dsum)	3.30 (1.67-6.52)	<0.001
log_2_(Vmax)	1.79 (1.36-2.34)	<0.001
log_2_(Vsum)	1.85 (1.41-2.43)	<0.001
Tumor & Lymph Node Status		
Number of tumors	0.99 (0.47-2.07)	0.98
Positive LN count (per 1 increase)	1.14 (1.08-1.22)	<0.001
Lymph Node Status (Positive vs Negative)	2.24 (0.74-6.82)	0.128
Pathological Characteristics		
Histological Grade (III vs I-II)	1.54 (0.58-4.10)	0.381
LVI (Positive vs Negative)	1.71 (0.68-4.35)	0.254
Ki-67 Index (High vs Low)	1.91 (0.74-4.94)	0.173
Molecular Subtype		0.012
HR+/HER2-	Reference	
HR+/HER2+	1.31 (0.27-6.30)	0.738
HR-/HER2+	5.73 (2.00-16.41)	0.001
HR-/HER2- **Surgery** BCS vs Mastectomy	4.45 (0.92-21.52) 0.24 (0.03-1.80)	0.063 0.084

Overall, all tumor size metrics demonstrated significant associations with DFS in univariable analysis.

### 3.4 Multivariable Cox regression analysis

In the multivariable Cox regression analysis, we included variables significantly associated with DFS in the univariable Cox regression analysis, excluding tumor size metrics. These variables included the positive lymph node (LN) count and molecular subtype. Clinically relevant factors such as LVI and Ki-67 index were also incorporated as covariates. We first constructed a baseline model based on the positive LN count (M0). After adding log_2_(Vsum), the M0 model combined with log_2_(Vsum) demonstrated superior performance across several key metrics (AIC = 138.42, C-index = 0.845, LRT P < 0.001) compared to the M0 model combined with log_2_(Dmax) (AIC = 143.01, C-index = 0.823, LRT P = 0.005). The M0 model combined with log_2_(Vmax) (AIC = 139.96, C-index = 0.847, LRT P < 0.001) showed slightly inferior goodness-of-fit compared to M0 model combined with log_2_(Vsum), whereas the M0 model combined with log_2_(Dsum) demonstrated the weakest predictive performance (AIC = 145.46, C-index = 0.796, LRT P = 0.018). We subsequently replaced the baseline model (M0) with molecular subtype, LVI, or the Ki-67 index and found that the model incorporating log_2_(Vsum) consistently achieved the best model fit, reflected by the lowest AIC, as well as the highest predictive accuracy, reflected by the highest C-index (**Table 4**).

**Table 4:** Multivariate Cox regression model comparison of disease-free survival prediction (*P* values are based on likelihood ratio tests).

Base model adjustment	Model	AIC	*P* value	C-index
Positive LN count	Base Model (M0)	149.06	—	0.771
	M0 + log_2_(Dmax)	143.01	0.005	0.823
	M0 + log_2_(Dsum)	145.46	0.018	0.796
	M0 + log_2_(Vmax)	139.96	<0.001	0.847
	M0 + log_2_(Vsum)	138.42	<0.001	0.845
Molecular Subtype	Base Model (M0)	157.23	—	0.697
	M0 + log_2_(Dmax)	147.19	<0.001	0.826
	M0 + log_2_(Dsum)	148.74	0.001	0.796
	M0 + log_2_(Vmax)	146.18	<0.001	0.848
	M0 + log_2_(Vsum)	145.22	<0.001	0.851
LVI	Base Model (M0)	162.89	—	0.602
	M0 + log_2_(Dmax)	152.26	<0.001	0.769
	M0 + log_2_(Dsum)	153.94	<0.001	0.744
	M0 + log_2_(Vmax)	149.43	<0.001	0.793
	M0 + log_2_(Vsum)	148.02	<0.001	0.802
Ki-67 Index	Base Model (M0)	162.73	—	0.554
	M0 + log_2_(Dmax)	151.37	<0.001	0.765
	M0 + log_2_(Dsum)	153.53	0.002	0.739
	M0 + log_2_(Vmax)	148.78	<0.001	0.784
	M0 + log_2_(Vsum)	147.68	<0.001	0.791

We further compared the multivariable Cox model that combined the positive LN count with log_2_(Dmax) (model 1) with the model that combined the positive LN count with log_2_(Vsum) (model 2). The model parameters are presented in **Table 5**. After 1,000 bootstrap resamples with replacement, model 2 achieved a corrected C-index of 0.842 (95% CI: 0.821–0.850), which exceeded model 1 (C-index = 0.822, 95% CI: 0.804–0.832). Schoenfeld residual testing confirmed that both models satisfied the proportional hazards assumption, as indicated by a non-significant P-value (P > 0.05) (**Table 6**). Across all model configurations, inclusion of log_2_(Vsum) consistently yielded superior model fit and discrimination.

**Table 5:** Parameter and performance comparison of key multivariate Cox regression. models.

Variable/metric	Model 1 HR (95% CI)	*P* value	Model 2 HR (95% CI)	*P* value
Positive LN count (per 1 increase)	2.611 (1.361–5.007)	0.004	1.773 (1.316–2.389)	<0.001
log_2_(Dmax)	1.125 (1.051–1.205)	<0.001	–	–
log_2_(Vsum)	–	–	1.128 (1.053–1.209)	<0.001
C-index	0.823	–	0.845	–
Bootstrap-corrected C-index (95% CI)	0.822 (0.804–0.832)	–	0.842 (0.821–0.850)	–

**Table 6:** Proportional hazards assumption test of key multivariate Cox models.

Model	Variable	χ² value	df	*P* value
Model 1	log_2_(Dmax)	0.20	1	0.65
	Positive LN count	0.49	1	0.48
	Global Test	0.79	2	0.68
Model 2	log_2_(Vsum)	1.47	1	0.23
	Positive LN count	0.32	1	0.57
	Global Test	1.60	2	0.45

### 3.5 Kaplan–Meier, ROC, and DCA analyses

**Figure 4:**
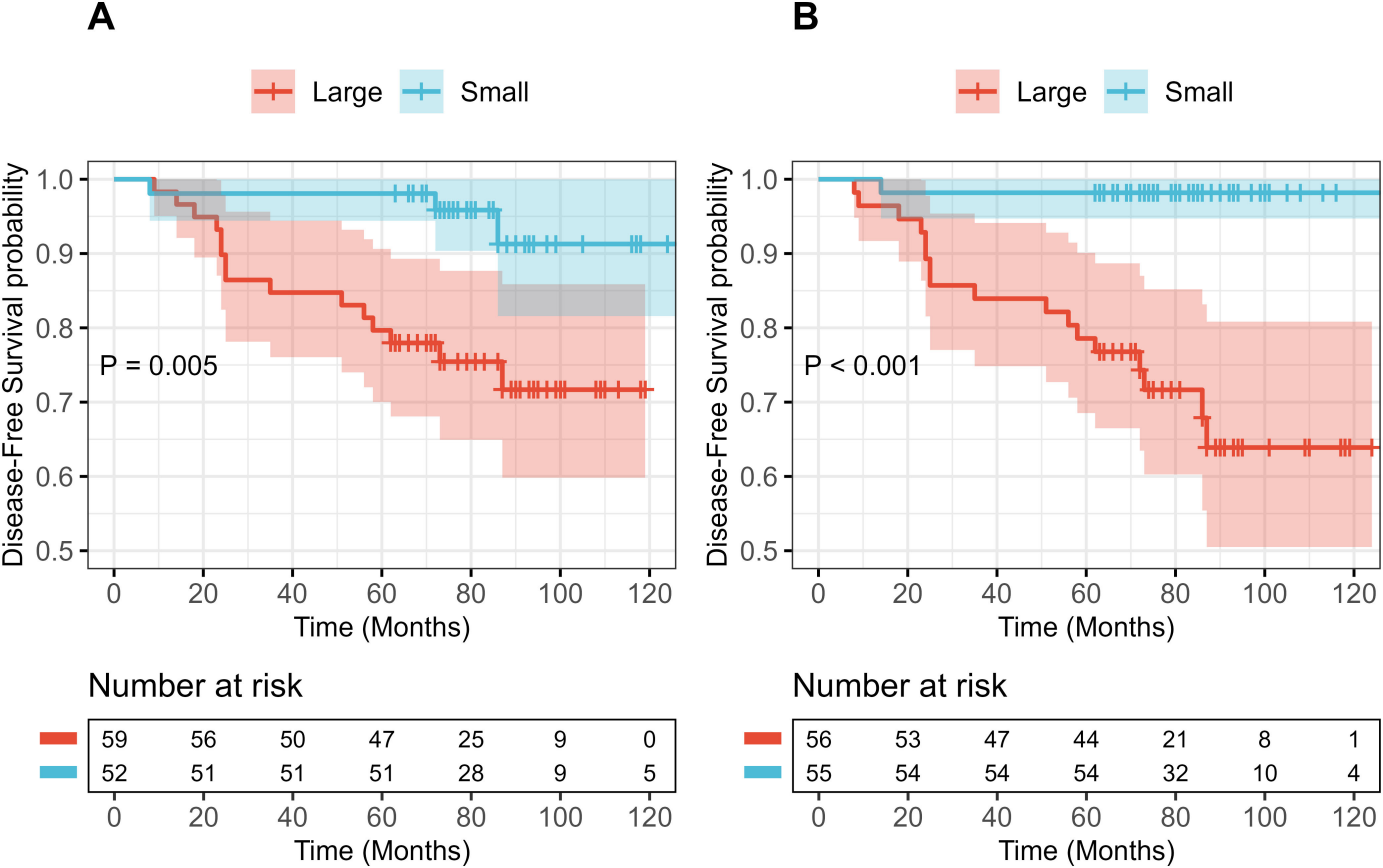
Kaplan–Meier curves for disease-free survival (DFS) stratified by tumor size. **(A)** Stratification by log_2_(Dmax) median (1.14) (log-rank P = 0.005). **(B)** Stratification by log_2_(Vsum) median (1.97) (log-rank P < 0.001).

Patients were divided into large and small groups based on the median values of log_2_-transformed sum of tumor volumes (log_2_(Vsum) = 1.97) and log_2_-transformed maximum tumor diameter (log_2_(Dmax) = 1.14), corresponding to median tumor volumes of 3.91 cm³ and diameter of 2.20 cm, respectively. Kaplan–Meier survival analysis revealed that log_2_(Vsum) provided clearer stratification of disease-free survival (DFS) (log-rank P < 0.001) compared with log_2_(Dmax) (log-rank P = 0.005) (**Figure 4**).

**Figure 5:**
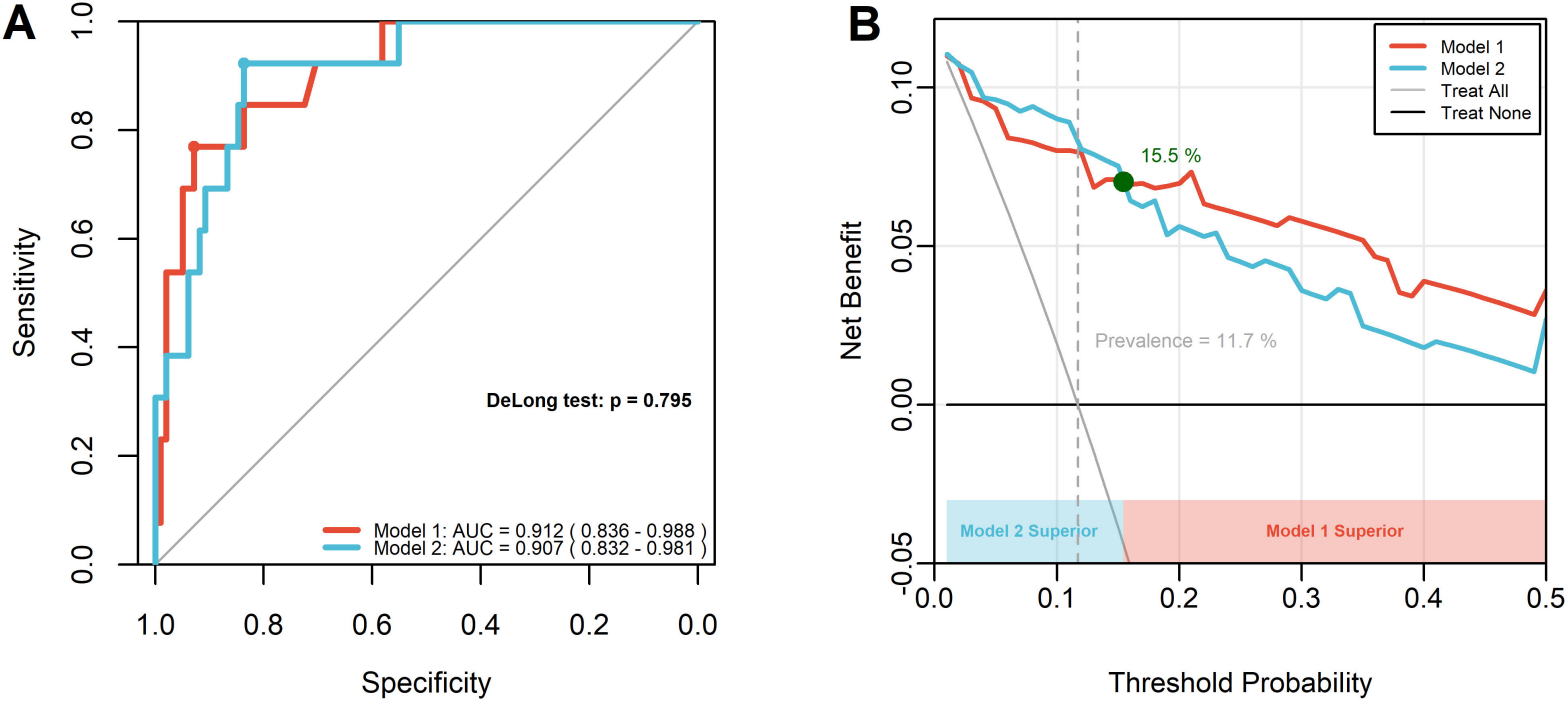
Comparison of Model 1 (positive LN count + log_2_(Dmax)) and Model 2 (positive LN count + log_2_(Vsum)) for predicting 5-year DFS. **(A)** ROC curves. Sensitivity and specificity were calculated using the Youden Index. **(B)** Decision curve analysis (DCA).

Using 5-year DFS as the binary endpoint, two multivariable logistic regression models were constructed: model 1 (positive LN count + log_2_(Dmax)) and model 2 (positive LN count + log_2_(Vsum)). Although receiver operating characteristic (ROC) curve analysis showed comparable area under the curve (AUC) values between model 1 and model 2 (0.845 vs. 0.832, DeLong P = 0.795), model 2 demonstrated higher sensitivity for predicting 5-year DFS (0.923 vs. 0.769) but slightly lower specificity compared with model 1 (0.837 vs. 0.923). Decision curve analysis (DCA) indicated that model 2 provided greater net clinical benefit at threshold probabilities below 15.5% (**Figure 5**). Collectively, these findings suggest that volumetric assessment provides additional prognostic information compared with diameter-based measurements.

## 4 Discussion

Multifocal/multicentric breast cancer (MMBC), characterized by multiple tumor foci within the breast, remains challenging in clinical practice, especially in accurately quantifying the overall tumor burden. Compared with unifocal breast cancer, MMBC patients are typically younger and exhibit higher rates of lymph node metastasis, recurrence, and distant metastasis, as well as a greater likelihood of undergoing mastectomy (3, 5, 7–10). In this cohort, patients aged ≤50 years (62.2%) were more frequent than those >50 years (37.8%); most patients had axillary lymph node metastasis (63.1%), 79.3% exhibited high Ki-67 expression, and 81.1% underwent mastectomy. However, existing evidence indicates that breast-conserving surgery is not contraindicated in MMBC, and its prognosis is not necessarily inferior to that of unifocal cases (3, 5). Notably, approximately 15–30% of MMBC patients display substantial inter-lesion molecular and clonal heterogeneity, which may influence tumor growth, metastatic potential, and treatment response (4, 11–13, 30). Consequently, relying solely on the largest lesion may underestimate the overall tumor burden, potentially affecting treatment decisions and prognosis. With advances in early diagnostic techniques, an increasing number of breast cancer patients are being diagnosed without distant metastases (18, 31). Accordingly, primary tumor size and nodal status have become key determinants for treatment planning and prognostic evaluation. Although the American Joint Committee on Cancer (AJCC) staging system defines T classification based on maximum tumor diameter (Dmax), this approach may underestimate the overall tumor burden in MMBC (19, 32).

Giuliano AE et al. noted that, due to limitations in measurement reproducibility, tumor diameter has long been considered a practical surrogate for tumor volume (19). With the widespread application of MRI, precise and reproducible tumor volume measurements have become feasible (33, 34). In this study, semi-automated 3D segmentation was performed using 3D Slicer, combining thresholding (range: 200–500 relative signal intensity units) with manual refinement to accurately delineate lesions. Interobserver agreement was excellent (Dice coefficient: 0.854 ± 0.08, ICC: 0.939–0.943), demonstrating high reproducibility. This approach offers greater precision than the ellipsoid approximation methods used in prior studies (6, 35–38). Several studies, including Fan Z et al. on 234 endometrial cancer patients, have confirmed that tumor volume is closely associated with pathological features and serves as an independent prognostic factor; similarly, Jeon SK et al. showed that MRI-based volumetry in hepatocellular carcinoma more accurately reflects tumor burden and improves prognostic performance (23, 24).

To our knowledge, this study is among the first to provide a systematic comparison between traditional diameter-based metrics and MRI-derived volumetric parameters in MMBC, demonstrating the added prognostic value of tumor volume for DFS and supporting its potential role in clinical decision-making. To ensure analytical rigor, a robust data-processing strategy was applied. Following the methodology reported by Zhang J et al., log_2_ transformation was applied to both diameter- and volume-based variables to enhance model interpretability (39). Under this transformation, the hazard ratios (HRs) can be directly interpreted in terms of risk changes associated with each doubling of the variable. For instance, an HR of 1.85 for log_2_(Vsum) suggests that doubling the sum of tumor volumes is associated with an 85% increase in recurrence and metastasis risk. This finding is consistent with the Gompertzian growth pattern of tumors, which approximates exponential expansion and enables more sensitive assessment of tumor burden (40, 41). Notably, the HR for diameter exceeded that for volume, as doubling the diameter increases the corresponding volume more than twofold.

In this study, tumor count was not significantly associated with DFS, suggesting that cumulative tumor burden rather than lesion number may be the primary determinant of prognosis. This likely reflects tumor heterogeneity. Multiple small lesions do not necessarily indicate greater biological aggressiveness when the overall tumor volume is limited, whereas fewer but larger or more aggressive lesions may confer a higher tumor burden. Therefore, lesion count alone is insufficient to capture the overall disease burden. Volumetric parameters such as Vsum may provide a more comprehensive assessment across all tumor foci. The absence of significant associations between DFS and factors such as the HR−/HER2− subtype, LVI, and lymph node positivity may be explained by several factors. The cohort included only patients without neoadjuvant therapy and without distant metastases, and was limited by a relatively small sample size and short follow-up duration. Furthermore, most patients had favorable luminal subtypes (88/111, 75.3%), whereas only 6 cases (5.4%) were classified as HR−/HER2−. In addition, immunohistochemical evaluation was performed for all lesions in only 40.5% of patients. In the remaining cases, only the dominant lesion was assessed, which may have underestimated inter-lesional heterogeneity and missed more aggressive subclones. These factors collectively may have attenuated the observed prognostic impact of established biomarkers in this cohort. Nevertheless, tumor size metrics, number of positive lymph nodes (LN), LVI, and molecular subtype are all established prognostic factors in breast cancer (6).

Given the strong correlations between tumor diameter and volume and the limited number of DFS events (18/111), a conservative multivariable Cox regression strategy was adopted, incorporating “one covariate plus one tumor-size variable” to minimize overfitting. As shown in **Table 3**, univariable Cox regression revealed that all tumor size metrics were significantly associated with DFS (P <0.05). In multivariable analyses, volumetric measures (Vsum and Vmax) remained independently prognostic even after adjusting for molecular subtype, Ki-67 index, and LVI, outperforming diameter-based metrics as indicated by lower AIC and higher C-index. Notably, log_2_(Vsum) demonstrated the most robust predictive value, suggesting that cumulative tumor volume provides a more stable prognostic indicator in MMBC. Koh HW et al. retrospectively analyzed 4,146 patients with T1–T2 invasive breast cancer and showed that tumor volume remained an independent predictor of recurrence-free survival (RFS) after adjusting for T stage by diameter (HR = 1.709; 95% CI: 1.161–2.516; P = 0.007) (42). Similarly, Hu J et al. found volumetric staging outperformed conventional T staging in nasopharyngeal carcinoma (43), with consistent observations in papillary thyroid, endometrial, and renal cell cancers (22, 44, 45). These findings also support that tumor volume better reflects tumor burden and provides superior prognostic value over tumor diameter. Specifically, the multivariable Cox model combining log_2_(Vsum) with positive lymph node count demonstrated the highest prognostic performance (AIC = 138.42, C-index = 0.845, P <0.001), followed by log_2_(Vmax) and log_2_(Dmax), whereas log_2_(Dsum) showed the lowest predictive efficacy. These findings indicate that the cumulative tumor volume across all lesions provides a more stable and reliable prediction of DFS than the diameter or volume of the largest lesion, with the sum of diameters performing the worst. Prior studies have proposed using the sum of tumor diameters (Dsum) for T staging (4, 20, 21); however, most evidence indicates that Dsum does not improve prognostic discrimination (6, 9, 32), consistent with our findings. This may reflect its tendency to overestimate the true tumor burden.

To ensure adequate sample size, patients were stratified by the median values of log_2_(Dmax) and log_2_(Vsum) for Kaplan–Meier survival analysis. The results demonstrated that log_2_(Vsum) provided superior DFS stratification compared with log_2_(Dmax) (log-rank P < 0.001 vs. 0.005), consistent with previous studies. Subsequently, multivariable logistic regression models were constructed for 5-year DFS prediction: Model 1 (log_2_(Dmax) + positive lymph node count) and Model 2 (log_2_(Vsum) + positive lymph node count). Receiver operating characteristic (ROC) analysis showed that Model 2 achieved substantially higher sensitivity for identifying high-risk patients (0.923 vs. 0.769), highlighting the superior clinical utility of volumetric assessment, in line with findings by Koh HW et al. (6, 29, 42). Decision curve analysis (DCA) further indicated that Model 2 conferred greater net clinical benefit when the threshold probability was below 15.5%. It should be noted, however, that postoperative adjuvant therapy data were not included, and therefore the clinical implications of these results should be interpreted with caution.

Based on long-term follow-up of 111 MMBC patients, this study is the first to demonstrate the potential clinical utility of volumetric metrics in this breast cancer subtype, filling a gap in understanding the relationship between tumor volume and prognosis in MMBC, and providing preliminary evidence for the broader applicability of volume-based prognostic assessment in solid tumors.

Several limitations should be noted. First, this study enrolled MMBC patients who underwent preoperative 3T MRI and had no distant metastasis, resulting in a relatively small number of DFS events. Although robustness was enhanced via simplified models, AIC comparison, and bootstrap resampling, the event-per-variable ratio (EPV = 9) remained below recommended thresholds, and the limited sample size precluded separate training and validation cohorts, potentially affecting model accuracy and generalizability. Second, as a single-center retrospective study, regional and institutional biases may exist, and only 40.5% of patients underwent immunohistochemical analysis of all lesions, potentially overlooking more aggressive subclones that could influence treatment decisions and outcomes. Multicenter studies are warranted to confirm generalizability. Third, MRI-based volumetric measurements require specialized software and experienced operators, limiting broader clinical implementation. Future research should investigate automated volumetric tools using more accessible imaging modalities, such as ultrasound, to improve applicability. Finally, the study did not explore the integration of tumor volume with radiomics, metabolomics, or proteomics. Incorporating volumetric parameters into multi-omics predictive frameworks may further refine prognostic assessment and support precision clinical decision-making in MMBC.

## 5 Conclusion

Tumor volumes derived from MRI-based three-dimensional reconstruction provide a more comprehensive assessment of the true tumor burden in multifocal/multicentric breast cancer (MMBC) and demonstrate superior predictive performance for disease-free survival (DFS) compared with conventional pathological diameters. These parameters may complement current T staging by providing a more comprehensive assessment of tumor burden. Given the single-center design, limited sample size, and short-term follow-up, these findings should be considered preliminary, and further multicenter prospective studies are warranted to confirm their generalizability and clinical utility.

## Data Availability

The raw data supporting the conclusions of this article will be made available by the authors, without undue reservation.
